# Elucidating polymorphs of crystal structures by intensity-based hierarchical clustering analysis of multiple diffraction data sets

**DOI:** 10.1107/S2059798323007039

**Published:** 2023-10-25

**Authors:** Hiroaki Matsuura, Naoki Sakai, Sachiko Toma-Fukai, Norifumi Muraki, Koki Hayama, Hironari Kamikubo, Shigetoshi Aono, Yoshiaki Kawano, Masaki Yamamoto, Kunio Hirata

**Affiliations:** a Life Science Research Infrastructure Group, RIKEN SPring-8 Center, 1-1-1 Kouto, Sayo-cho, Sayo-gun, Hyogo 679-5198, Japan; b Structural Biology Division, Japan Synchrotron Radiation Research Institute, 1-1-1 Kouto, Sayo-cho, Sayo-gun, Hyogo 679-5148, Japan; c Graduate School of Science and Technology, Nara Institute of Science and Technology, 8916-5 Takayama-cho, Ikoma, Nara 630-0192, Japan; d Japan Agency for Medical Research and Development-Core Research for Evolutional Science and Technology (AMED-CREST), Tokyo 100-0004, Japan; e Institute for Molecular Science, National Institutes of Natural Sciences, 5-1 Higashiyama, Myodaiji, Okazaki, Aichi 444-8787, Japan; f Exploratory Research Center on Life and Living Systems, National Institutes of Natural Sciences, 5-1 Higashiyama, Myodaiji, Okazaki, Aichi 444-8787, Japan; Stanford University, USA

**Keywords:** polymorph analysis, high-data-rate macromolecular crystallography, hierarchical clustering analysis, automated structure analysis

## Abstract

In macromolecular structure determination using X-ray diffraction from multiple crystals, the presence of different structures (structural polymorphs) necessitates the classification of the diffraction data for appropriate structural analysis. Hierarchical clustering analysis (HCA) is a promising technique that has so far been used to extract isomorphous data, mainly for single-structure determination. Although in principle the use of HCA can be extended to detect polymorphs, the absence of a reference to define the threshold used to group the isomorphous data sets (the ‘isomorphic threshold’) poses a challenge. Here, unit-cell-based and intensity-based HCAs have been applied to data sets for apo trypsin and inhibitor-bound trypsin that were mixed post data acquisition to investigate the efficacy of HCA in classifying polymorphous data sets. Single-step intensity-based HCA successfully classified polymorphs with a certain ‘isomorphic threshold’. In data sets for several samples containing an unknown degree of structural heterogeneity, polymorphs could be identified by intensity-based HCA using the suggested ‘isomorphic threshold’. Polymorphs were also detected in single crystals using data collected using the continuous helical scheme. These findings are expected to facilitate the determination of multiple structural snapshots by exploiting automated data collection and analysis.

## Abstract 2

Single-step intensity-based hierarchical clustering is demonstrated to allow the detection of structural polymorphs in diffraction data sets obtained from multiple crystals. By splitting data sets collected using a continuous helical scheme into several chunks, both inter-crystal and intra-crystal polymorphs can successfully be analyzed.

## 1. Introduction

The automation and acceleration of data collection at macromolecular crystallography (MX) beamlines of synchrotron-radiation facilities are yielding massive amounts of X-ray diffraction data. Such highly efficient, high-yield data acquisition is now becoming a major trend in MX; namely, high-data-rate MX (HDRMX; Bernstein *et al.*, 2020 ▸). HDRMX has been realized by the availability of highly brilliant X-ray beams (Ursby *et al.*, 2020 ▸; Sanchez-Weatherby *et al.*, 2019 ▸; Hirata *et al.*, 2013 ▸), the fast readout of detectors (Casanas *et al.*, 2016 ▸), rapid sample-exchange robots (Murakami *et al.*, 2020 ▸; Papp *et al.*, 2017 ▸; Martiel *et al.*, 2020 ▸; Nurizzo *et al.*, 2016 ▸) and the application of automated measurement schemes (Zander *et al.*, 2015 ▸; Hirata *et al.*, 2019 ▸; Basu *et al.*, 2019 ▸; Bowler *et al.*, 2016 ▸). Furthermore, data reduction and structure analysis of the obtained data sets has also been automated in various pipelines (Yamashita *et al.*, 2018 ▸; Winter & McAuley, 2011 ▸; Wojdyr *et al.*, 2013 ▸; Winter, 2010 ▸; Monaco *et al.*, 2013 ▸; Von­rhein *et al.*, 2011 ▸; Incardona *et al.*, 2009 ▸; Wang *et al.*, 2022 ▸). Finally, data-management systems, including user interfaces for viewing these data, have been developed so that experimenters can seamlessly manage and easily interpret the results of analyses from large amounts of data (Yamada *et al.*, 2013 ▸; Delagenière *et al.*, 2011 ▸; Fisher *et al.*, 2015 ▸).

Recently, structure determination using multiple crystals has been facilitated by various developments that include the automation and acceleration of data collection. In structure determination using microcrystals, especially from lipid cubic phase (LCP) crystals of membrane proteins, structures are normally determined using the small-wedge synchrotron crystallography (SWSX) approach. In the SWSX approach, the total oscillation range is reduced but the number of photons per oscillation width is increased. Since each wedge data set covers only part of reciprocal space, tens to hundreds of data sets must be measured from many microcrystals that are mounted in different orientations (Cherezov *et al.*, 2007 ▸; Rosenbaum *et al.*, 2007 ▸). Finally, the acquired data sets are merged and used to determine the structure. In the case of serial femtosecond crystallography (SFX; Barends *et al.*, 2022 ▸) or serial synchrotron rotation crystallography (SSROX; Gati *et al.*, 2014 ▸; Hasegawa *et al.*, 2017 ▸), a much larger number of images is required because every frame covers only a small portion of reciprocal space, posing challenges in data collection and analysis. Here, the automation of data collection and analysis is crucial and has provided opportunities to expand the target and achieve structure determination of diverse protein samples (Healey *et al.*, 2021 ▸).

During structural analysis using multiple crystals, it is crucial to select data sets that are sufficiently isomorphous. For example, if non-isomorphous data sets are merged in SAD phasing, it greatly complicates the search for the heavy-atom positions (Giordano *et al.*, 2012 ▸; Baba *et al.*, 2021 ▸). Hierarchical clustering analysis (HCA) is an approach that has successfully been utilized to extract highly isomorphous data sets from multiple crystals. This approach has been implemented in the program *BLEND* (Foadi *et al.*, 2013 ▸) that conducts unit-cell-based HCA, while HCA based on the correlation of diffraction intensities (also referred to as ‘intensity-based HCA’) has been realized in the program *ccCluster* (Santoni *et al.*, 2017 ▸). The automatic data-processing pipeline *KAMO* implements both types of HCA.

Even when a structure can be solved using a single crystal, there are benefits to using multiple crystals to analyze the structure. One benefit is in terms of attainable resolution. The resolution of a given structure can be improved as the number of data sets for merging increases because the signal-to-noise ratio of weak diffraction spots is improved. Another benefit is linked to the analysis of polymorphs. Physiologically meaningful structural polymorphs have been found by classifying diffraction data from many crystals. Most previous studies used HCA to extract highly isomorphic data for single-structure determination. However, some recent studies have demonstrated that HCA can be a powerful tool to classify structural polymorphs (Nguyen *et al.*, 2022 ▸; Soares *et al.*, 2022 ▸). In principle, HCA does not require any prior information on how many data clusters (*i.e.* the number of ‘polymorphs’ in the context of MX) are involved in the entire data set. In practice, however, there are generally two ways to interpret the results from HCA. One approach determines a threshold for the degree of ‘isomorphism’, and clustered data below this threshold are considered to underlie the same structure. The other approach decides the number of data clusters prior to analysis. Since it is impossible to know how many polymorphs are involved in the entire data set, the former approach is desirable. However, since there is no reference to decide an appropriate threshold (‘isomorphic threshold’) at present, it is necessary to analyze the merged data sets in each cluster exhaustively and the results should be interpreted case by case. Therefore, we investigated the feasibility of assigning an ‘isomorphic threshold’ which can be used to select candidate data clusters where polymorphs may be identified.

Furthermore, we also investigated the usefulness of applying HCA to data collected using the helical scheme to capture both inter-crystal and intra-crystal structural polymorphs. The helical scheme, strictly referred to as the ‘continuous helical scheme’, was originally developed as a data-collection scheme to avoid severe radiation damage (Flot *et al.*, 2010 ▸). In contrast to a conventional single-point oscillation scan, crystals are translated during data collection in the helical scheme. Therefore, the dose for each crystal volume can be considered to be constant. Since each frame is acquired from a different point in the crystal, splitting the full data set into several partial data sets (chunks) could be useful to observe structural differences that are present in the crystal by processing each chunk individually and classifying them by HCA. Indeed, using this approach, structure determination was possible even from heterogeneous crystals (Katoh *et al.*, 2020 ▸).

In this paper, we sought to determine the ‘isomorphic threshold’ using *in silico* mixed data sets consisting of two different high-resolution data sets from standard test protein samples (trypsin). This threshold was then applied to two representative protein samples (the nuclear transport receptor transportin-1 and the [NiFe]-hydrogenase maturation factor HypD) to evaluate whether the suggested threshold suitably classifies polymorphs.

## 2. Materials and methods

### 2.1. Preparation of apo and inhibitor-bound trypsin crystals

Bovine pancreatic trypsin (molecular weight of approximately 24 kDa; Fujifilm Wako Pure Chemicals) was dissolved in 25 m*M* HEPES pH 7.0 with 5 m*M* CaCl_2_ to a concentration of 30 mg ml^−1^. The precipitant solution was 30%(*w*/*v*) PEG 3350, 0.1 *M* Tris–HCl pH 8.5, 0.2 *M* Li_2_SO_4_. Crystallization was performed at 293 K by the sitting-drop vapor-diffusion method using MRC-II plates (SWISSCI) and crystals of 200 µm in size appeared within a few days. Several crystals were harvested before adding the compound to obtain data for the apo form. The crystals were soaked in cryoprotectant, which consisted of 10%(*v*/*v*) ethylene glycol mixed with the crystallization buffer, and were then cryocooled in liquid nitrogen (Yamane *et al.*, 2011 ▸).

The inhibitor compound was directly added to the droplet on the crystallization plates using an Echo 650 acoustic liquid handler (Beckman Coulter). In this study, the following inhibitors were used (Supplementary Fig. S1): 4-methoxy­benzamidine and 5-chlorotryptamine (hereafter referred to as ‘benzamidine’ and ‘tryptamine’, respectively). Each inhibitor was added to the crystallization droplet at a final concentration of 10 m*M* containing 10%(*v*/*v*) dimethyl sulfoxide (DMSO). After addition of the inhibitor, the crystallization plate was placed at 293 K for an hour to allow sufficient diffusion of the inhibitor into the crystals. Inhibitor-bound trypsin crystals were fished out from the crystallization plate, cryoprotected in a similar manner to apo trypsin crystals and cryocooled in liquid nitrogen. The DMSO concentration and the incubation time were determined based on the results of a preliminary study. In brief, several series of crystals with different DMSO concentrations and incubation times were prepared using apo trypsin. Data were then collected from these crystals to identify conditions under which the diffraction quality was not significantly degraded.

### 2.2. Diffraction data collection, data processing and structure determination of trypsin

All diffraction data for trypsin crystals were collected on BL32XU at SPring-8 using the automated data-collection system *ZOO* (Hirata *et al.*, 2019 ▸). Data were obtained from four crystals of apo, benzamidine-bound and tryptamine-bound trypsin. All data sets were acquired using a continuous helical scheme with 360° of oscillation and the following experimental parameters: oscillation width, 0.1°; exposure time, 0.02 s; beam size, 10 µm (horizontal) × 15 µm (vertical); wavelength, 1 Å; average dose per crystal volume, 10 MGy; detector, EIGER X 9M (Dectris); temperature, 100 K. The *KUMA* module of *ZOO* automatically estimates the attenuation factor from the measured crystal size and the designated dose value (Hirata *et al.*, 2019 ▸). The rotation axis of the goniometer is horizontal to the ground. Basically, the irradiation vector was set in the apparent long-axis direction of the crystal, which roughly coincides with the rotation axis, in the *ZOO* helical scheme.

The data obtained were automatically processed by *XDS* (Kabsch, 2010 ▸) in *KAMO* (Yamashita *et al.*, 2018 ▸). Subsequently, automated structural analysis was performed using *NABE* (Matsuura *et al.*, unpublished work), an automated structural analysis pipeline currently under development at the SPring-8 MX beamlines. *NABE* provides an interface to manage all of the merged data in clustering results generated by *KAMO* by summarizing the data statistics and the electron-density map. *NABE* runs *DIMPLE* (Wojdyr *et al.*, 2013 ▸) on the data processed by *KAMO* using the given template model for molecular replacement. If the amino-acid residues and atom names are specified for a site of interest, *NABE* automatically generates pictures of the protein model and the resultant electron-density maps (2*F*_o_ − *F*_c_ and *F*_o_ − *F*_c_ maps) around the selected site using *Coot* (Emsley *et al.*, 2010 ▸), *Raster*3*D* (Merritt & Bacon, 1997 ▸) and *ImageMagick* (https://imagemagick.org). The pictures are not a static snapshot but a GIF image that rotates, making it easier to see the surrounding environment, including the depth direction, which is difficult to perceive from a static image. After the above processes are complete, *NABE* returns an HTML report that tabulates the resolution of the obtained data, *R*_free_, the *B* factor *etc.* from the results of *KAMO* and *DIMPLE*, along with the GIF image for each data set. When multiple data sets are used, for example during SWSX measurements, *KAMO* classifies the diffraction data using HCA and then merges them into multiple clusters. Subsequently, *NABE* automatically performs an instantaneous data analysis for all of these merged data sets.

Here, using the report from *NABE*, we inspected the electron-density map around the inhibitor-binding site of trypsin and confirmed that *F*_o_ − *F*_c_ density for inhibitors was not observed in apo trypsin. In contrast, *F*_o_ − *F*_c_ density for each inhibitor was observed for the benzamidine-bound and tryptamine-bound trypsin data sets (Supplementary Fig. S1).

### 2.3. Clustering analysis using data from apo and inhibitor-bound trypsin

In HCA, ‘isomorphism’ between data sets is represented as a vertical ‘distance’ in the dendrogram. Isomorphous data sets are linked by smaller distances, while other, more distantly related data sets are linked by longer distances. Supposing that there are several structural polymorphs in the multiple data sets, each polymorph will make a cluster consisting of data sets within a certain ‘threshold’ (referred to as an ‘isomorphic threshold’) in the dendrogram. In actual data, the number of polymorphs involved in the obtained data set is unpredictable. Therefore, two apparently different data sets were used to investigate an ‘isomorphic threshold’ for identifying polymorphs. For this purpose, we used high-resolution data sets from apo trypsin and trypsin bound to two different inhibitors.

Two different parameter-based HCAs are implemented in *KAMO*: one is unit-cell-based and the other is intensity-based HCA. *KAMO* performs unit-cell-based HCA using *BLEND*. Intensity-based HCA is performed based on calculation of the CC by the *cctbx* (Grosse-Kunstleve *et al.*, 2002 ▸) method *miller_array.correlation.coefficient* and grouping by the *SciPy* (Virtanen *et al.*, 2020 ▸) method *cluster.hierarchy.dendrogram*. The structural changes of protein molecules in the crystal may possibly affect the unit-cell constants or diffraction intensities. These two different parameter-based HCAs are somewhat different in terms of isomorphism. In unit-cell-based HCA the classification is based on the isomorphism of the unit-cell parameters, which reflects a more macroscopic aspect rather than the protein structures. In contrast, intensity-based HCA can detect much smaller structural changes.

Here, we examined how two different data sets are classified by the two different parameter-based HCAs: unit-cell-based and intensity-based. To evaluate whether splitting the data collected by the helical scheme enables polymorph analysis within the same crystals, the collected data were divided into 30° chunks and individually processed using *KAMO* with the split_data_by_deg=30.0 option. The test data set for clustering analysis was prepared by mixing the same number of diffraction data sets from two different structures. In this study, 360° of data collected using the helical scheme were used after division into 30° chunks. Here, we used 48 30° chunks for each structure, corresponding to data sets from four crystals. The two parameter-based HCAs were applied to *in silico* mixed data sets consisting of 96 chunks from the following combinations of two data sets (48 + 48 chunks): (i) apo and benzamidine-bound trypsin and (ii) benzamidine-bound and tryptamine-bound trypsin. HCA and data merging were carried out using *KAMO* (*kamo.auto_multi_merge*) in the following scheme. Prior to HCA, *KAMO* selects the data sets used in the merging step (referred to as ‘pre-processing’). Firstly, the equivalent data group is selected based on *P*1 symmetry. The selected data sets are then filtered based on the unit-cell constant using Tukey’s criterion. HCA is performed for the filtered data list, and merged data are generated for each cluster. *KAMO* rejects data on a frame-by-frame and a data set-by-data set basis based on crystallographic statistics in the three cycles of data merging. The electron-density map for the merged data at each cluster is depicted to evaluate the effect of data contamination. Molecular replacement was performed with the template model (PDB entry 3rxa). Evaluation of the electron-density map was performed using *NABE*.

In general, the definition of ‘isomorphism’ between data sets and the linkage method used to calculate the distance between clusters contribute significantly to the resultant dendrogram from HCA. For intensity-based HCA, the correlation coefficient (CC) of intensity is used as an indicator of isomorphism. A different definition of distance is used in *KAMO* and *ccCluster* as a default setting: (1 − CC)^1/2^ is used in *KAMO* (Yamashita *et al.*, 2018 ▸), while (1 − CC^2^)^1/2^ is used in *ccCluster* (Santoni *et al.*, 2017 ▸). In this study, we used (1 − CC^2^)^1/2^ (hereafter referred to as *d*_CC_) based on preliminary investigations of the following distance definitions available in *KAMO*: 1 − CC, (1 − CC)^1/2^ and (1 − CC^2^)^1/2^. For the linkage method, the ‘Ward’ linkage is often used, as it is empirically less prone to causing ‘chain effects’ and ‘inversions’ of the dendrogram (Murtagh & Legendre, 2014 ▸). *BLEND* also adopts the Ward method for the same reason (Foadi *et al.*, 2013 ▸), while *ccCluster* uses the ‘average’ linkage method instead. In the intensity-based HCA implemented in *KAMO*, the Ward linkage is utilized as the default setting. Based on the results with seven linkage methods available in the *SciPy* module (Supplementary Section S1 and Fig. S2), we used the Ward linkage in this study. In the Ward linkage, the closest cluster is selected by minimizing the increase in the variance when joining the clusters. When merging a pair of single data sets, the Ward distance can be considered to be synonymous with *d*_CC_, so that the CC value can directly be calculated from the value of the Ward distance. In contrast, when merging clusters containing multiple data sets, the distance between these clusters is determined based on the data contained in the cluster. Accordingly, the Ward distance does not directly give the CC value for each data set inside the clusters. For example, a Ward distance of 0.6 does not mean that the CC value for each pair of data sets equals 0.8.

### 2.4. Determination of the isomorphic threshold from observed/simulated data

Based on the results of HCA of trypsin *in silico* mixtures, we hypothesized that the presence of polymorphic structures could be detected by the Ward distance threshold on the vertical axis of the dendrogram. If there are multiple clusters below this ‘isomorphic threshold’, each cluster is considered to be a different structure. However, the absolute values of the Ward distance generally increase with the number of data sets and they also increase when the CC distributions among different structures are diverse. Therefore, it would not be useful to derive the absolute Ward distance threshold directly from the dendrogram of observed trypsin data as a general-purpose index. Therefore, we simply define the isomorphic threshold using the maximum Ward distance (*W*_0_ in Supplementary Fig. S3) of the entire system as 

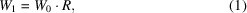

where *W*_0_ is the maximum Ward distance, *W*_1_ is the isomorphic threshold and *R* is a constant to be determined here (a ratio with a magnitude between 0 and 1).

The ‘isomorphic threshold’ is defined as the larger Ward distance between two branches when two structures can be classified (*W*_1_ in Supplementary Fig. S3). To determine *R* in equation (1), the simulation was performed with the following steps. Based on the apo and benzamidine-bound trypsin data sets, we firstly modeled the intensity CC distribution for all of the three combinations. Assuming that there are multiple data sets and two structures are included, we created the CC matrix for HCA using CCs that follow the probability density of our model (Supplementary Fig. S3). We performed HCA from the CC matrix and evaluated whether the two structures could be classified as their original labels on the dendrogram.

Firstly, we checked whether the simulation successfully reproduced the dendrograms obtained experimentally. Since *W*_0_ and *W*_1_ can be obtained from the dendrogram in the HCA simulation, they were simply compared with the observed values. Next, the CC model parameters were changed from the initial model to make classification increasingly difficult, and calculations were performed stepwise until the final condition where classification was no longer possible. The number of data sets was varied from 100 to 1000, each with half of two different structural labels. The *R* calculated from *W*_1_/*W*_0_ and the score in each HCA were plotted for each parameter to determine a suitable *R* to calculate the isomorphic threshold in the intensity-based HCA. The details of the simulation are described in Supplementary Section S2.

### 2.5. Application to representative sample 1: polymorph analysis of the nuclear transport receptor transportin-1 in complex with a nuclear localization signal peptide

To evaluate whether the ‘isomorphic threshold’ suggested from the investigations of trypsin data sets is effective, HCA was carried out on representative sample data sets with the proposed ‘isomorphic threshold’.

Transportin-1 (Trn1) is one of the nuclear transport receptors which recognizes a nuclear localization signal (NLS) sequence harbored in cargo proteins and brings them into the nucleus. Detailed information about sample preparation of the Trn1–NLS peptide complex (with molecular weights of approximately 98 and 2.5 kDa, respectively) is described in Supplementary Section S3. We describe it briefly here. The Trn-1 Δloop mutant was produced using an *Escherichia coli* expression system. Purification of Trn1 was performed using Glutathione Sepharose and anion-exchange columns followed by size-exclusion chromatography. The NLS peptide of Trn1 (Eurofins) was dissolved in purification buffer (110 m*M* potassium acetate, 200 m*M* HEPES–KOH, 10 m*M* DTT). The Trn1–NLS peptide complex (hereafter referred to as the ‘Trn1–peptide complex’) was prepared by mixing 5 mg ml^−1^ Trn1Δloop and 5 m*M* NLS peptide. Trn1–peptide complex crystals were obtained using a crystallization condition consisting of 0.5 *M* sodium potassium phosphate pH 5.0. The obtained crystals were cryocooled in liquid nitrogen after cryoprotection with a 30%(*w*/*v*) glycerol-containing reservoir solution. Diffraction data were automatically collected on BL32XU at SPring-8 using *ZOO*. From each of the four crystals, 720° of rotation data were acquired using a continuous helical scheme. The other experimental parameters were identical to those used for the trypsin crystals: oscillation width, 0.1°; exposure time, 0.02 s; beam size, 10 µm (horizontal) × 15 µm (vertical); wavelength, 1 Å; average dose/crystal volume, 10 MGy; detector, EIGER X 9M (Dectris). The *KUMA* module of *ZOO* automatically estimated the attenuation factor from the measured crystal size and the designated dose value. The obtained 720° of data were split into 30° chunks and hierarchical clustering was applied. Using *NABE*, the electron-density map was compared with the peptide-free structure model as a template model for MR. The peptide-free model was prepared prior to the clustering analysis by the following procedure: phasing by molecular replacement using *Phaser* (McCoy *et al.*, 2007 ▸) with a template model (PDB entry 5yvi) followed by iteration of refinement using *phenix.refine* (Liebschner *et al.*, 2019 ▸) and manual model building using *Coot*.

### 2.6. Application to representative sample 2: polymorph analysis of the [NiFe]-hydrogenase maturation factor HypD

HypD is one of the maturation factors of [NiFe]-hydrogenase and can form a complex with other maturation factors (Muraki *et al.*, 2019 ▸). The C360S variant of HypD from *Aquifex aeolicus* (hereafter referred to as *Aa*HypD-C360S, molecular weight of approximately 42 kDa) was produced using an *E. coli* expression system and was purified using a cation-exchange column with a sodium chloride gradient and size-exclusion chromatography. *Aa*HypD-C360S crystals were obtained using 16%(*w*/*v*) polyethylene glycol 3350, 0.1 *M* citrate buffer pH 5.6, 1 m*M* dithiothreitol as a reservoir solution. The obtained crystals were cryocooled in liquid nitrogen after cryoprotection. Diffraction data were automatically collected on BL45XU at SPring-8 using *ZOO*. From each of the six crystals, 360° of rotation data were acquired using a continuous helical scheme. The other experimental parameters were as follows: oscillation width, 0.1°; exposure time, 0.02 s; detector, PILATUS3 6M (Dectris). The *KUMA* module of *ZOO* automatically estimated the attenuation factor from the measured crystal size and the designated dose value. The obtained 360° of data were split into 30° chunks and hierarchical clustering was applied. The data were merged into largely separated data clusters that should correspond to structural polymorphs. Evaluations of the electron-density map were carried out by *NABE*. Before analysis by *NABE*, a template model (PDB entry 2z1d) was prepared using *Phaser* *REFMAC*5 (Murshudov *et al.*, 2011 ▸) and *Coot*.

## 3. Results and discussion

### 3.1. Test study: classification of different trypsin data sets

To investigate whether HCA can classify polymorphic data sets, *in silico* mixed data sets were prepared which consist of 48 chunks from each of two different (apo and benzamidine-bound) trypsin data sets, resulting in a total of 96 chunks that were submitted to *KAMO*. The resolution of each chunk was approximately 1.2 Å. Each chunk contained about 50 000 reflections; the overall completeness was about 40% and the space group was *P*2_1_2_1_2_1_. *KAMO* rejected 13 chunks during pre-processing as described in Section 2, leaving 83 chunks for HCA (Supplementary Table S1). Reflections up to 1.50 Å resolution were used to calculate the correlation between the data sets in intensity-based HCA.

#### 3.1.1. Classification of diffraction data for apo trypsin and benzamidine-bound trypsin

**Figure 1:**
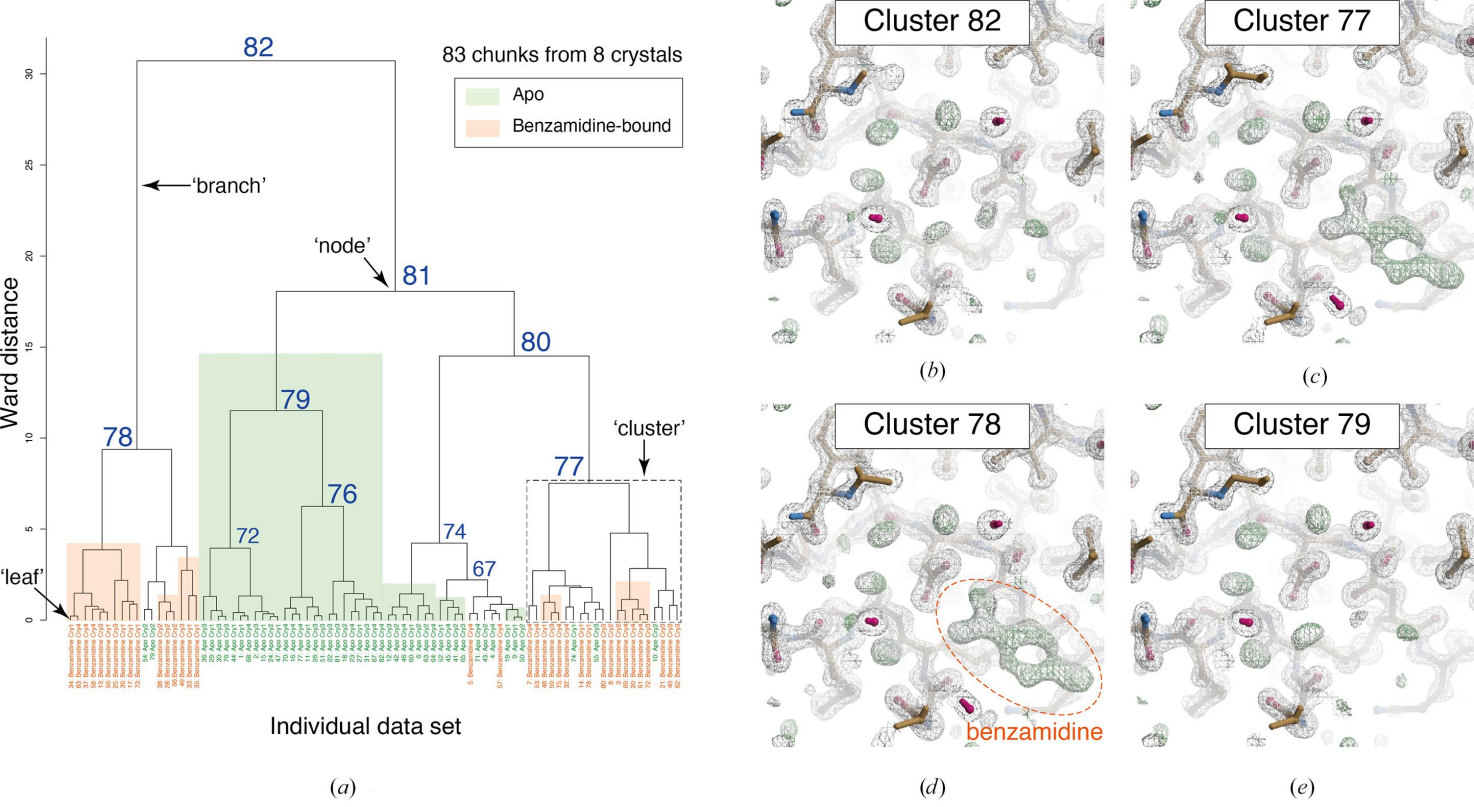
Results of unit-cell-based HCA of *in silico* mixed data sets containing apo and benzamidine-bound trypsin. (*a*) Resultant dendrogram. The definitions of ‘node’, ‘branch’ and ‘cluster’ used in this study are illustrated in the dendrogram. Cluster numbers and data labels are depicted on the resultant dendrogram from unit-cell-based HCA. The blue numbers indicate the cluster numbers at each node. Chunks for apo trypsin are shaded green and those for benzamidine-bound trypsin are colored orange. (*b*)–(*e*) Electron-density maps around the inhibitor-binding site of trypsin in different clusters. The contour level of the 2*F*_o_ − *F*_c_ map (gray mesh) is 1.0σ and that of the *F*_o_ − *F*_c_ map (green mesh) is 3.0σ. The maps were generated by *Coot* in the *NABE* system.

Unit-cell-based and intensity-based HCAs were applied to *in silico* mixed data sets including apo and inhibitor-bound trypsin (Figs. 1 ▸ and 2 ▸). In both HCA results, the merged data for the top cluster (Cluster 82) exhibited no significant *F*_o_ − *F*_c_ density corresponding to benzamidine at 3.0σ, even though chunks of benzamidine-bound trypsin data sets were included in the mixed data set (Supplementary Table S2). This may be explained by the fact that the number of apo trypsin data sets in the merged data was larger than that for benzamidine-bound trypsin as a consequence of pre-processing by *KAMO* before HCA.

The largest linear cell variation (LCV) value (Foadi *et al.*, 2013 ▸), a characteristic quantity for unit-cell variation, was 0.59%. Due to slight differences in the unit-cell constant between apo and benzamidine-bound trypsin (Supplementary Fig. S4), unit-cell-based HCA did not succeed in fully separating apo and benzamidine-bound trypsin data sets (Fig. 1 ▸). This implies that unit-cell-based HCA is less sensitive to a small lattice change such as that found between the apo and benzamidine-bound trypsin crystal forms. In practical cases, well classified clusters should be inferred from the electron-density map since there is no label for polymorphs. Accordingly, we investigated the electron-density map obtained from merged data in each cluster. The two different data sets appeared to be separated at the first branch of the dendrogram (Clusters 78 and 81) based on the electron-density map. Although both apo and benzamidine-bound chunks are involved in these clusters, data contamination cannot be distinguished using the electron-density map. Due to the rejection of outlier data sets during the merging process by *KAMO*, some of the contaminating data chunks were eliminated. For example, although Cluster 78 has two apo trypsin and 16 benzamidine-bound trypsin chunks immediately after clustering, ten chunks containing only benzamidine-bound trypsin data sets were merged as the final data set, resulting in the presence of clear electron density for benzamidine (Supplementary Table S2 and Fig. 1 ▸). In most of the other cases, the final merged data still contained some contamination even after outlier rejection (Supplementary Table S2). For instance, the merged data in Cluster 77 consisted of two apo trypsin and 11 benzamidine-bound trypsin chunks. However, the small amount of contamination did not affect the resulting electron-density map (Fig. 1 ▸). Approximately 15% contamination did not affect the electron-density map resulting from the dominant data set in this test case.

**Figure 2:**
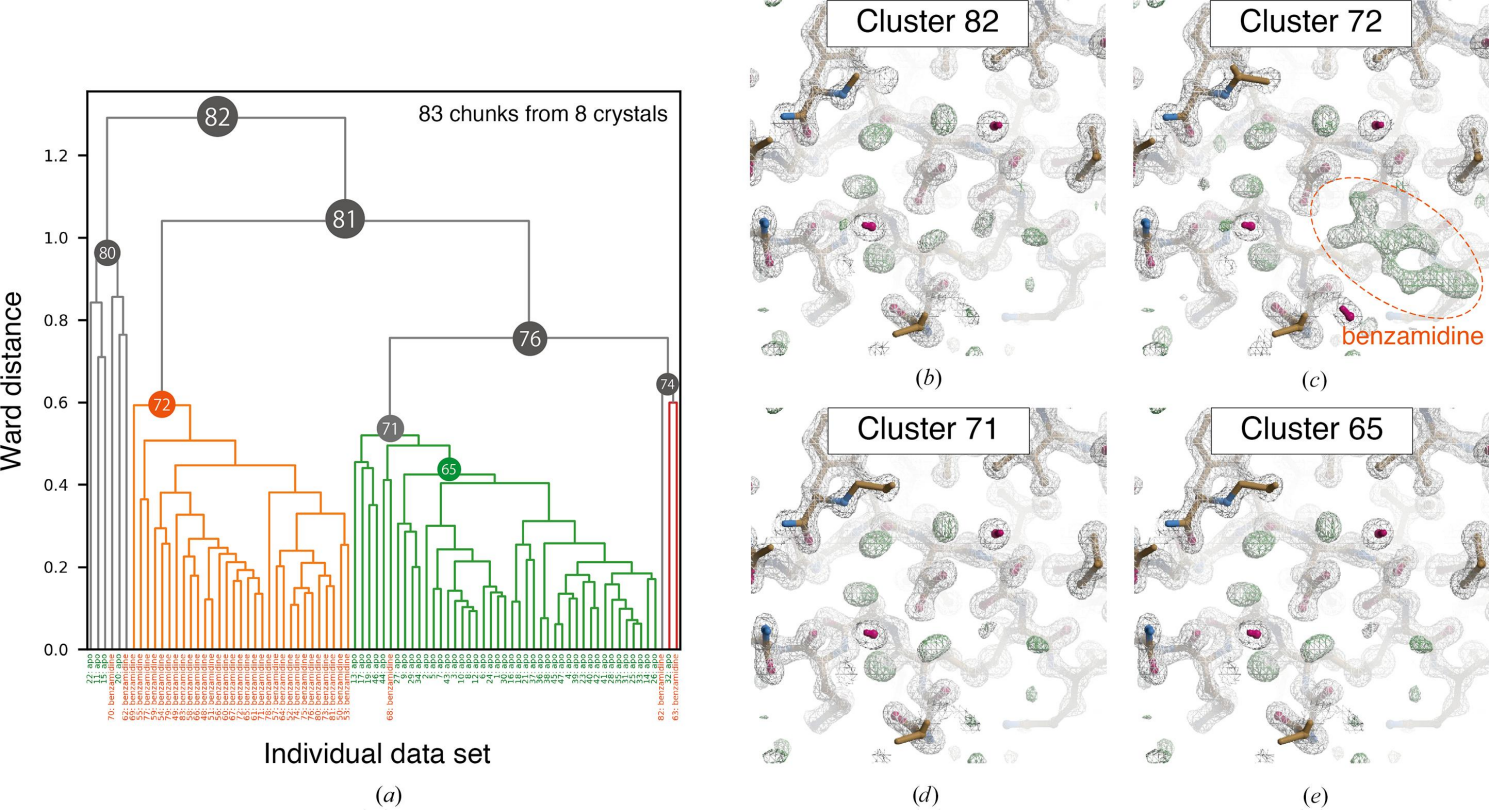
Results of intensity-based HCA on *in silico* mixed data sets containing apo and benzamidine-bound trypsin. (*a*) Resultant dendrogram. Data labels are shown at the bottom: green, apo trypsin; orange, benzamidine-bound trypsin. The color threshold for the dendrogram is set to 0.6. (*b*)–(*e*) Electron-density maps around the inhibitor-binding site obtained from the merged data in different clusters. The contour levels of the 2*F*_o_ − *F*_c_ map (gray mesh) and the *F*_o_ − *F*_c_ map (green mesh) are 1.0σ and 3.0σ, respectively. The maps were generated by *Coot* in the *NABE* system.

In contrast to the above-described results, intensity-based HCA (Fig. 2 ▸) succeeded in classifying the two mixed data sets completely (Clusters 71 and 72). Clear *F*_o_ − *F*_c_ density for benzamidine was observed in Cluster 72, while no significant *F*_o_ − *F*_c_ density for benzamidine was observed in other clusters at 3.0σ. Cluster 71 had only one benzamidine-bound chunk, and there are two possible reasons why this chunk was involved in the apo trypsin cluster: either the occupancy of benzamidine was low or the data quality was poor. Considering the former possibility, the data obtained from a selected benzamidine-bound crystal was divided into four 90° chunks and occupancy refinement of benzamidine was performed with *REFMAC*5. The resulting occupancies of 97%, 93%, 97% and 92% were not significantly different, suggesting that the ligand occupancy was likely to be constant over the whole crystal volume. With regard to the latter possibility, automated helical data collection from large crystals often results in significantly low diffraction power at both ends of the crystal. This point is further discussed in Section 3.1.4.

In intensity-based HCA, the CC is key information that has an influence on the results of classification. In CC calculation, common reflections up to the specified resolution are used. To investigate the resolution dependence of the CC distance (*d*_CC_) value, intensity-based HCA was performed at several resolution cutoffs (Supplementary Fig. S5). Despite decreasing the cutoff resolution for CC calculation to 3.5 Å, intensity-based HCA successfully classified apo and benzamidine-bound trypsin data chunks. Accordingly, the resolution dependence of the CC calculation appeared to be insignificant as far as we investigated. The successful sorting of apo and benzamidine-bound data chunks demonstrates that intensity-based HCA can be effective in disentangling heterogeneous data chunks, even if the differences in cell dimensions are too small to be classified by unit-cell-based HCA.

#### 3.1.2. Classification of two different inhibitor-bound trypsin data sets

Next, we tested HCA-based classification on data sets obtained from crystals of trypsin with two different inhibitors with different skeletal formulas (Supplementary Fig. S1) but that share the same binding site: benzamidine and tryptamine.

**Figure 3:**
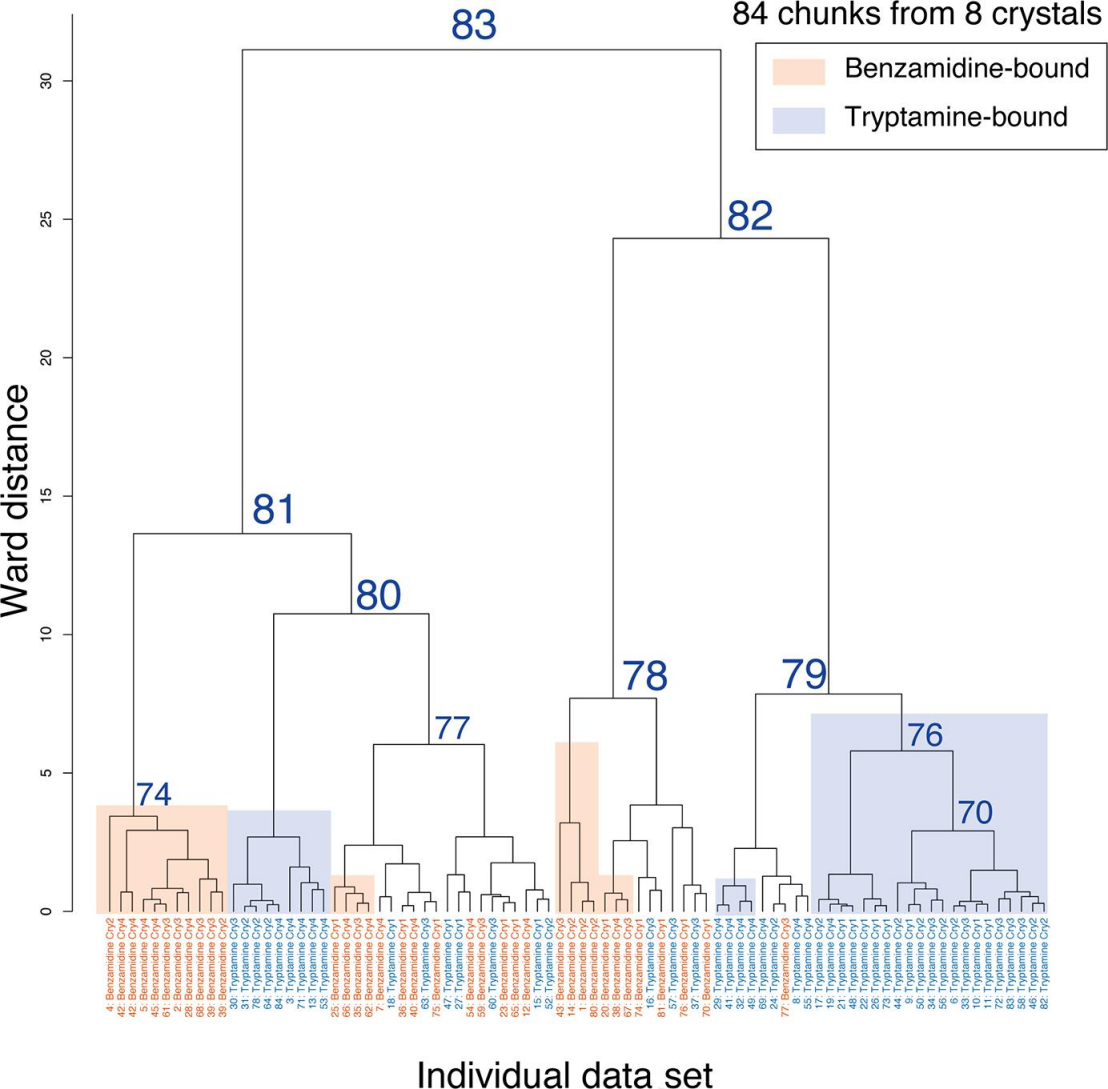
Dendrogram from unit-cell-based clustering of mixed data sets including benzamidine-bound and tryptamine-bound trypsin. Cluster numbers and data labels are depicted on the resultant dendrogram from unit-cell-based HCA. To evaluate the clustering effect, each cluster leaf is shaded according to the original data set: orange, benzamidine-bound trypsin; blue, tryptamine-bound trypsin. The number labeled at each cluster node indicates the cluster number output from *BLEND*.

Although clusters of homogenous data sets can be found for some clusters (for example Clusters 74 and 76), the data for the two different inhibitors could not be successfully sorted by unit-cell-based HCA (Fig. 3 ▸). The largest LCV value was 0.75%, which is slightly larger than that for apo and benzamidine-bound trypsin. This implies that a larger LCV value of 0.75% is still insufficient to classify the different trypsin data sets using unit-cell-based HCA. Based on the electron-density map (Supplementary Fig. S6), the two different data sets appeared to be roughly separated at the first branch (Clusters 81 and 82). However, the electron-density map in Cluster 80 clearly exhibited a mixture of both types of inhibitor-bound data. It appears to be difficult to distinguish the base skeletal structure from the electron-density map. The merged data consisted of six benzamidine-bound chunks and 14 tryptamine-bound chunks, resulting in approximately 30% contamination of the dominant data set (Supplementary Table S3). In Cluster 77, the merged data consisted of nine benzamidine-bound chunks and four tryptamine-bound chunks, with about 31% contamination of the prevailing data set. Based on these results, approximately 30% contamination by a minor data set might be a tolerable upper limit to obtain an electron-density map that allows initial model building.

**Figure 4:**
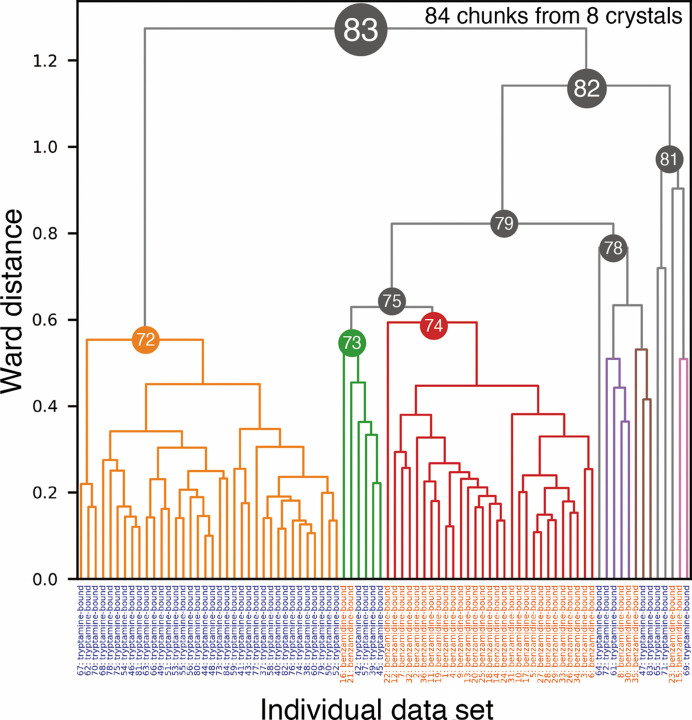
Dendrogram from intensity-based HCA of mixed data sets including benzamidine-bound and tryptamine-bound trypsin. The data labels at the bottom are in orange for benzamidine-bound trypsin and in blue for tryptamine-bound trypsin.

Intensity-based HCA (Fig. 4 ▸) successfully sorted the data chunks into two homogenous data sets (Clusters 72 and 74), although two inhibitor-bound data sets were mixed in some small clusters in the right branch (Clusters 73, 78 and 81). The electron-density maps obtained at Clusters 74 and 72 exhibited clear *F*_o_ − *F*_c_ densities derived from benzamidine and tryptamine at 3.0σ, respectively (Supplementary Fig. S7).

#### 3.1.3. Classification of apo trypsin and two inhibitor-bound trypsin data sets

As shown in the previous sections, two different data sets were classified using single-step intensity-based HCA. However, more than three structural polymorphs may be found in practical cases. For example, when clustering is applied to data obtained in a time-resolved experiment, there may be more than three intermediates before and after the intermediate of interest. To test such a case, intensity-based HCA was applied to *in silico* mixed data sets containing all three of the different trypsin data sets used in this study, namely apo, benzamidine-bound and tryptamine-bound trypsin data sets.

The results showed that intensity-based HCA classified the three different data sets almost perfectly (Supplementary Fig. S8), while unit-cell-based HCA did not classify these data sets well (Supplementary Fig. S9). In the first branch in Supplementary Fig. S8, one cluster contains only tryptamine-bound chunks (Cluster 115) while the other mainly consists of apo and benzamidine-bound chunks (Cluster 130). From further clustering of the latter cluster, the homogeneous data cluster for apo trypsin appeared at Cluster 114 and that for benzamidine-bound trypsin appeared at Cluster 116. During separation, some small clusters (Clusters 119, 120 and 128) appeared. These clusters are considered to be outliers because the number of chunks involved is smaller and the distances between the chunks are relatively large compared with the other clusters. Although some inhibitor-bound chunks were mixed into the dominant apo trypsin cluster (Cluster 114), this is possibly due to lower inhibitor occupancies or relatively low-quality data.

#### 3.1.4. The ‘isomorphic threshold’ suggested from investigations on the observed trypsin data sets

The CC values for the data sets in the clusters for *in silico* mixed trypsin (apo and benzamidine-bound trypsin; Section 3.1.1) were 0.93 and 0.94 for the clusters of apo trypsin (Cluster 71) and benzamidine-bound trypsin (Cluster 72) data sets, respectively. This result suggests that HCA is effective in the classification of such small structural changes. Therefore, we investigated the change in CC on a structural change of a certain volume of the protein moiety. The change in CC value was examined for a rotation of the partial or whole trypsin molecule (the full length comprises 223 amino acids) without any changes in the unit-cell constants (Supplementary Fig. S10). It was found that even the relatively significant conformational change of a 5° rotation of the terminal ten-amino-acid helix resulted in a CC decrease of 0.015 compared with the original structure. When a quarter of the trypsin residues (57 amino acids) were rotated, a 5° rotation resulted in a CC change of approximately 0.030. The simulation also proved that larger CC changes occur if the whole molecule is rotated.

For the 30° chunks for apo (referred to as ‘apo’) and benzamidine-bound (referred to as ‘benz’) trypsin used in the present study, a histogram of the *d*_CC_ values obtained for each combination of chunks is illustrated in Supplementary Fig. S11. The distribution of *d*_CC_ for homogeneous pairs of apo chunks was centered at around 0.2, whilst the *d*_CC_ obtained between heterogeneous data for apo and benz trypsin had a distribution shifted slightly to the right at around 0.25 (CC ≃ 0.97).

The histograms showed that even among homogeneous data, some portions of the combination exhibited a high *d*_CC_ value of greater than 0.6 (Supplementary Fig. S11). The heat map of *d*_CC_ values between all pairs of data sets revealed that there are some data sets that do not have any correlation against almost all of the data (Supplementary Fig. S12). These data were mostly chunks emanating from the tip of the crystals. Since the selection of equivalent data sets and unit-cell-based filtering are performed prior to clustering, these chunks are similar to the other chunks, at least with respect to the unit-cell parameters. The chunks at the tip of crystals did not significantly affect the structural analysis, as discussed in the previous section; therefore, the calculation of CC may be not reliable (for example 〈*I*/σ(*I*)〉 or the resolution limit for used intensities should be carefully considered during the calculation of CC). However, all of the data classified by HCA were included in the main distribution with a *d*_CC_ of around 0.2, indicating that the main *d*_CC_ distribution is important for accurate classification by HCA.

As described in Section 2.4 and Supplementary Section S2, numerical simulations were performed to determine an ‘isomorphic threshold’ from apo and benz trypsin data sets. The observed median values for the CC_apo–apo_, CC_benz–benz_ and CC_apo–benz_ distributions were 0.978, 0.970 and 0.962, respectively, with corresponding standard deviations of 0.020, 0.019 and 0.017, respectively. Only data satisfying *d*_CC_ < 0.4 were used to characterize the prominent peak of the CC distribution for these statistics. The distributions of CCs and fitted curves are shown in Supplementary Fig. S13(*a*).

The HCA simulations for apo and benz trypsin showed perfect classification without contamination of the dendrogram (Supplementary Fig. S13*b* ). After repeating 100 calculations, the mean and standard deviation of *W*_1_ for classifying the two different structures were 0.61 and 0.03, respectively. The result demonstrates that our simulation roughly reproduces the observed data.

**Figure 5:**
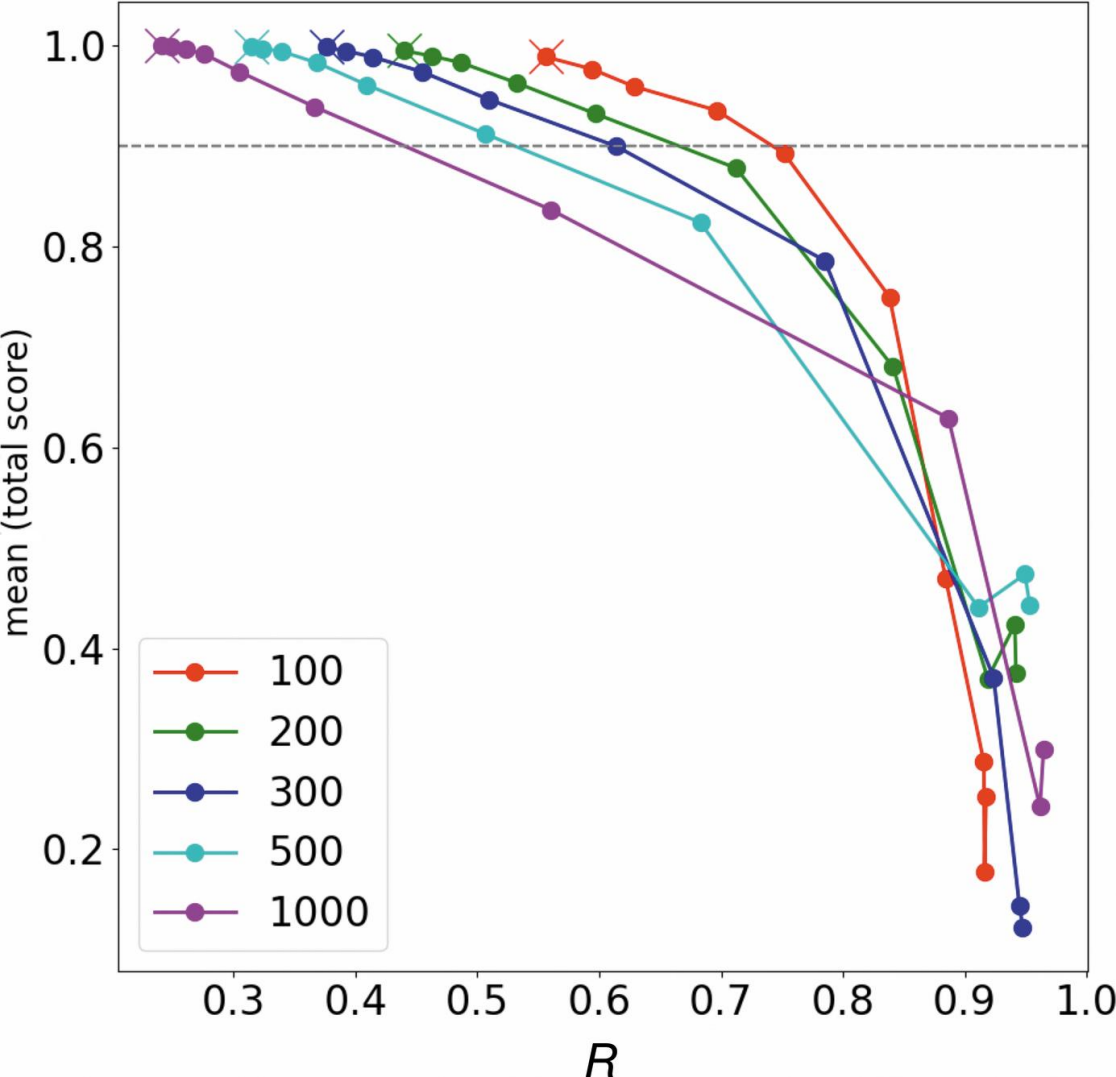
*R* in equation (1) and classification score in our HCA simulations (details are described in Section 2.4 and Supplementary Section S2). The line plots illustrate the scores assuming 100, 200, 300, 500 and 1000 data sets. The lowest *R* in each plot is shown as a cross mark and is the result of HCA using the original CC model (Supplementary Fig. S13*a* ). HCA simulations were performed with the CC_apo–benz_ and CC_benz–benz_ positions gradually approaching each other in ten steps, making classification more difficult.

In the next step, the validated model was used to perform HCA simulations by modifying its parameters to make it difficult to classify the two structures. Fig. 5 ▸ displays the relationship between the ratio *R* and the HCA simulation scores. The lowest *R* in each plot, shown as a ‘cross mark’, is the result of HCA using the original model. From there, as classification becomes difficult, *R* increases and the score worsens. An *R* greater than 0.7 worsens the classification score for all plots except for 1000 data sets. We regard the score threshold for success in classification as 0.9 (dotted line in Fig. 5 ▸); 0.6–0.7 can then be a reasonable *R* for several hundred data sets. Based on these results, we assumed that the ‘isomorphic threshold’ for polymorphism detection in intensity-based HCA can be calculated by multiplying *W*_0_ by 0.6–0.7.

By using the assumed ratio, classifications in the observed intensity-based HCA were examined. For the apo/benz trypsin case, 0.77–0.90, the highest value of the dendrogram in Fig. 2 ▸ (1.29) multiplied by 0.6–0.7, was used as the isomorphic threshold. As a result, Clusters 71, 72 and 74 were candidates for structural polymorphs, but Cluster 74 was not a complete data set and could not be used for structural analysis. For the two ligand-bound trypsins (Fig. 4 ▸) the isomorphic threshold is 0.76–0.89, which classifies Clusters 72, 75 and 78 as candidates for structural polymorphs. Although Cluster 73 is contaminated, Cluster 75 shows a benzamidine-bound structure in the electron-density map in Supplementary Fig. S7. A similar result is found in intensity-based HCA on a mixed data set including apo and benzamidine-bound trypsin. In Supplementary Fig. S8, the isomorphic threshold is about 0.96–1.1, and Clusters 115, 123 and 125 are candidates for polymorphs. Of these, Clusters 115, 114 and 116 remain after excluding the obvious outlier clusters. Our isomorphic threshold proved to be a good indicator for classifying polymorphism in all trypsin cases.

The investigations on trypsin data sets indicated that intensity-based HCA can successfully classify structural polymorphs. The results also suggested that single-step intensity-based HCA could be sufficient when variation in the unit-cell constant is small; for example, when the largest LCV value is less than 1%. In the dendrogram from intensity-based HCA on trypsin test cases (Figs. 2 ▸ and 4 ▸ and Supplementary Fig. S8), the same data sets appeared to be clustered within our isomorphic threshold. Based on the results, polymorphs could be identified by grouping the data sets using a single step of intensity-based HCA with the suggested ‘isomorphic threshold’. Since multiple steps of clustering (Nguyen *et al.*, 2022 ▸) decrease the number of data sets in the final step by filtering the data during the clustering, fewer steps of clustering may be helpful when the total number of data is limited.

### 3.2. Applications to representative samples

To evaluate whether a single step of intensity-based HCA with our suggested ‘isomorphic threshold’ is useful for the detection of polymorphs in practical samples, we performed intensity-based HCA and structure analysis on two representative examples: the Trn1–peptide complex and *Aa*HypD-C360S. Here, we selected clusters that satisfy our threshold, together with the completeness of the merged data being high enough to allow further structural analysis.

#### 3.2.1. Diffraction data classification and polymorph analysis of the Trn1–peptide complex

Diffraction data of the Trn1–peptide complex were obtained using a helical scheme for 720° rotation. Each data set was divided into 30° chunks, yielding 96 chunks from four crystals. The resolution for each chunk was in the range 3–4 Å. The number of reflections in each 30° chunk was approximately 60 000, the overall completeness was about 25% and the space group was *C*2. Reflections up to 3.67 Å resolution were used to calculate the correlation between the data sets in intensity-based HCA.

**Figure 6:**
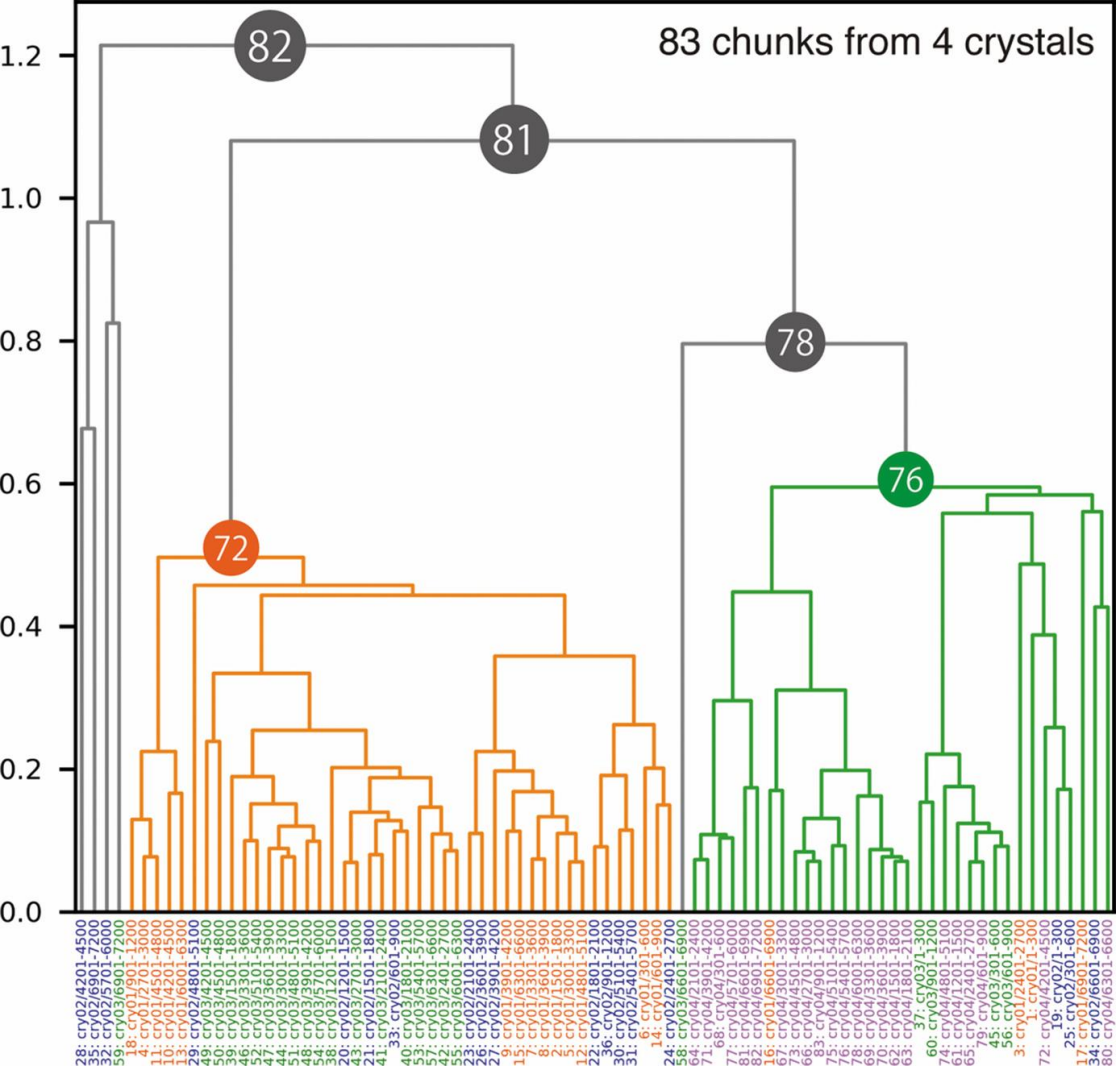
Dendrogram from intensity-based HCA for the Trn1–peptide complex. Data labels are shown at the bottom and are colored by crystals. Clusters 72 and 76 were used for further structural analysis because these two clusters appeared to have different structures (polymorphs).

The obtained chunks were subjected to intensity-based HCA using *KAMO* (Fig. 6 ▸). Although some outliers were observed, two major clusters (Clusters 72 and 76) were selected for further structural analysis.

**Figure 7:**
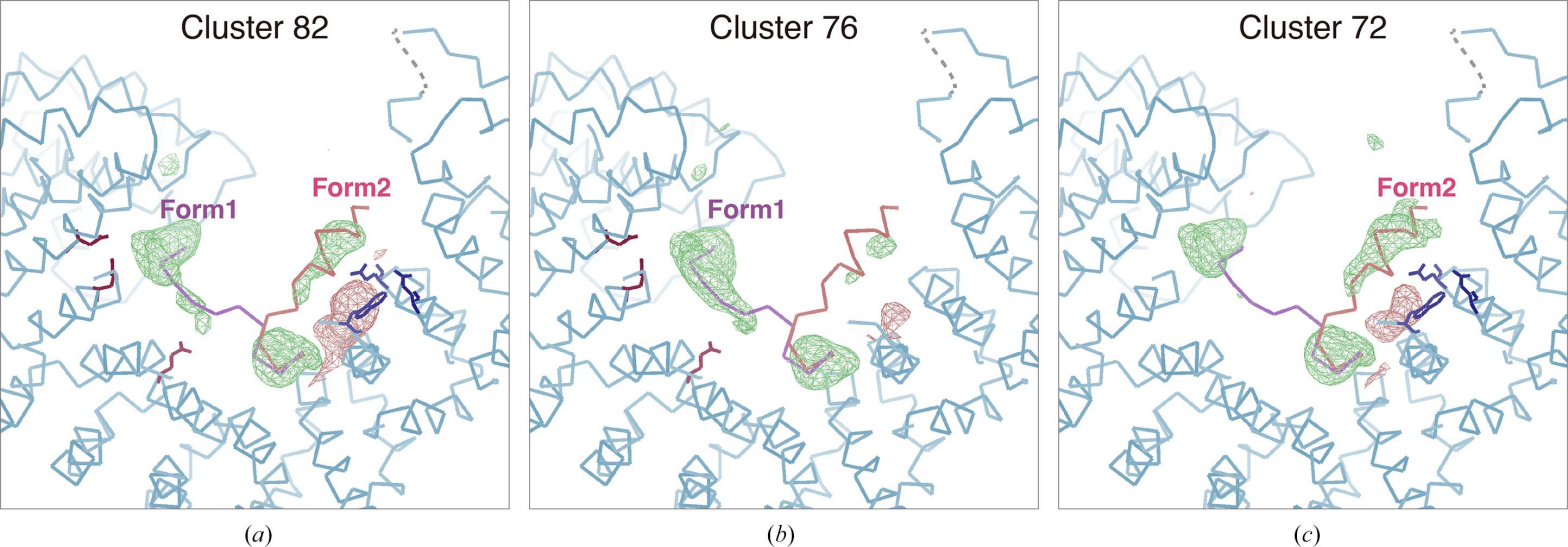
Peptide-omitted *F*_o_ − *F*_c_ maps from different nodes resulting from intensity-based HCA for the Trn1–peptide complex. *F*_o_ − *F*_c_ maps calculated from merged data at (*a*) Cluster 82, (*b*) Cluster 76 and (*c*) Cluster 72 are depicted. The contour level for each figure is set to 3.0σ. The binding peptide was omitted during map calculation. The figures were generated by *Coot*.

The electron-density maps from these two nodes were significantly different (Fig. 7 ▸). There were two peptide binding forms: one without any secondary structure (Form 1) and one with an α-helix (Form 2). These two peptide binding forms were also observed when all chunks were merged (Cluster 82). However, the *F*_o_ − *F*_c_ map for each peptide binding form appeared to be less clear than the clustering results (Clusters 72 and 76). Although both peptide binding forms appeared in each node, the occupancies seemed to be different. The *F*_o_ − *F*_c_ map indicated that Form 1 was dominant in Cluster 76 while Form 2 was dominant in Cluster 72. The isomorphic threshold values range from 0.73 to 0.85, and looking at the dendrogram with these values, Clusters 72 and 76 are candidates for structural polymorphs, consistent with the above results.

The existence of two peptide binding forms was supported by the results of biochemical experiments. As illustrated in Fig. 7 ▸, some acidic amino-acid residues were found around the *F*_o_ − *F*_c_ density of the NLS peptide in both binding forms. Since NLS generally consists of basic amino-acid residues, this is in line with the cargo-recognition mechanism which is achieved by electrostatic interaction. Triple mutations in each peptide binding form (two Glu residues and one Asp residue for Form 1 and two Glu residues and one Trp residue for Form 2) caused a decrease in peptide binding. Thus, both peptide binding forms are physiologically important for the function of Trn1.

Since the variation of unit-cell constants for the Trn1–peptide complex was more significant than that of trypsin (Supplementary Fig. S14), we also applied unit-cell-based HCA (Supplementary Fig. S15). The largest LCV value for the Trn1–peptide complex was 4.03%, which was significantly higher than that for trypsin (0.59%). The obtained electron-density maps seemed different (Supplementary Fig. S16), as observed in the results from intensity-based HCA (Fig. 7 ▸). However, the clustering result was slightly different. Form 1 dominant nodes appeared in the left branch (Clusters 73 and 78), while both forms were observed in the right branch (Clusters 77 and 79). The Form 2 dominant node was not found in the results from unit-cell-based HCA. Accordingly, single-step intensity-based HCA seemed to classify the data better in this case even though the cell variation is significant.

Although the resolution was relatively low (lower than 3 Å) and the space group was not highly symmetric (*C*2) in the case of the Trn1–peptide complex, intensity-based HCA was adequate to identify two different peptide binding modes. The results imply that polymorph analysis can even be performed on data sets that exhibit relatively low resolution and low-symmetry space groups.

Another interesting finding is that the dominant peptide binding form differed even though the crystals were obtained in the same crystallization drop. Furthermore, some crystals had both Form 1 dominant and Form 2 dominant chunks (*i.e.* intra-crystal variation). Mapping the clustering results revealed that Form 1 dominant chunks were mostly found at the tips of the crystals (Supplementary Fig. S17). This result implies that polymorphs not only in different crystals (inter-crystal) but also in the same crystals (intra-crystal) are discernible by collecting diffraction data via the continuous helical scheme, significantly expanding the possibilities of polymorph analysis.

#### 3.2.2. Diffraction data classification and polymorph analysis of *Aa*HypD-C360S

Using a helical scheme, diffraction data sets for *Aa*HypD-C360S were acquired from six crystals, applying 360° rotation per crystal. Each data set was divided into 30° chunks, yielding 72 chunks from six crystals. The resolution for each chunk was approximately 1.6 Å. There were about 100 000 reflections in each 30° chunk, the overall completeness was about 45% and the space group was *P*2_1_2_1_2_1_. For intensity-based HCA, reflections up to 2.79 Å resolution were used to calculate the correlation between the data sets.

**Figure 8:**
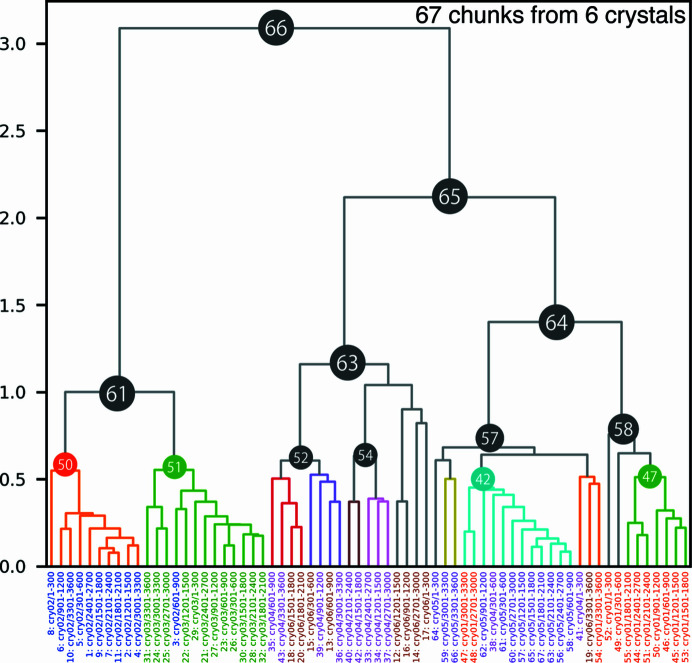
Dendrogram from intensity-based HCA on the *Aa*HypD-C360S variant. Data labels are shown at the bottom, colored by crystals. Due to the significant deviation among the data sets, some clusters were not satisfied with sufficient completeness for further analysis. Accordingly, Clusters 52 and 54 were selected based on the results with a threshold of 0.8. Each data cluster consists of chunks from one or two crystals, indicating that inter-crystal differences are more prominent than intra-crystal differences in *Aa*HypD-C360S.

**Figure 9:**
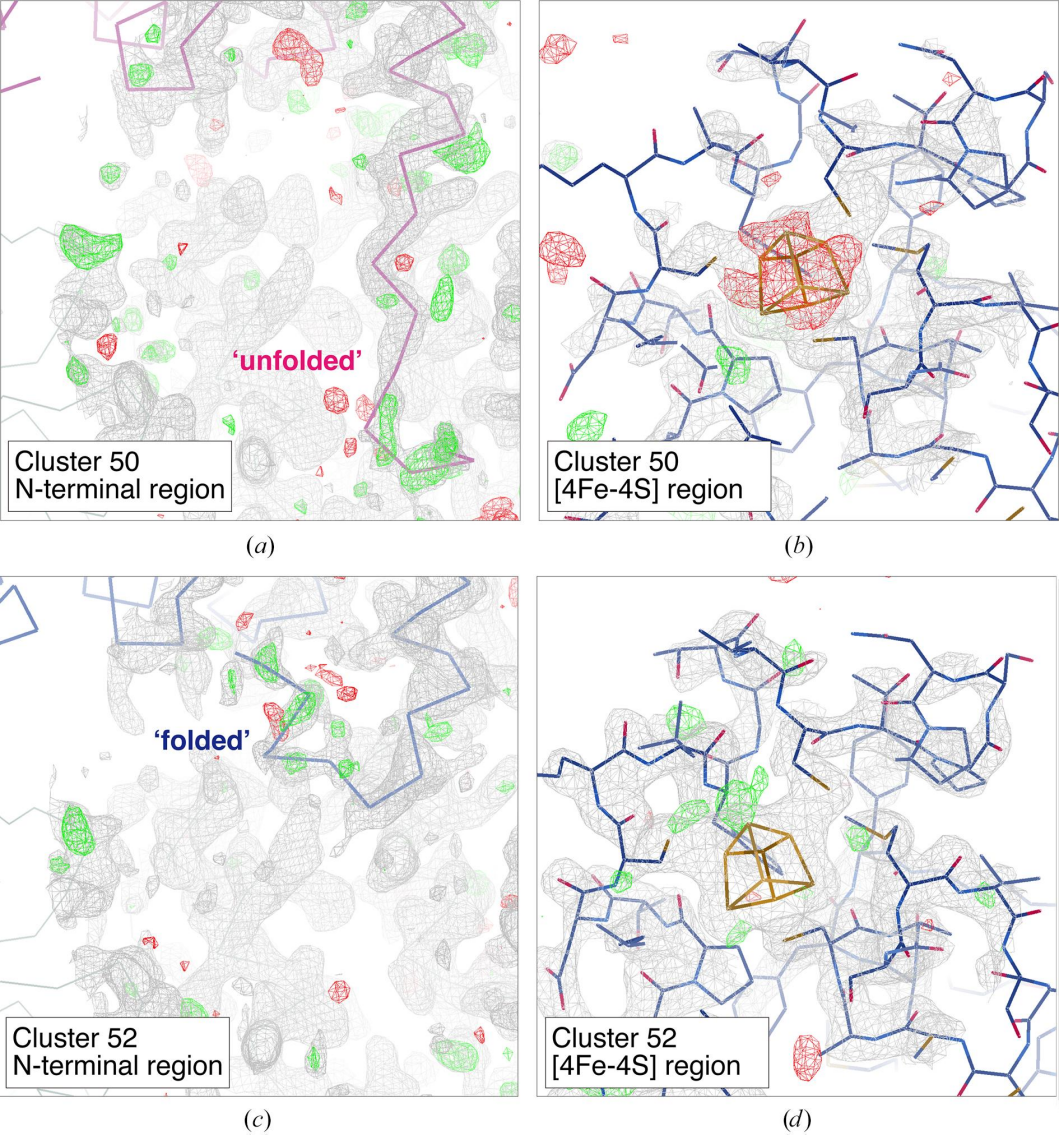
Electron-density maps around the N-terminal region and [4Fe–4S] cluster obtained from merged data at different clusters: (*a*, *b*) Cluster 50 and (*c*, *d*) Cluster 52. The contour level for the 2*F*_o_ − *F*_c_ map (gray mesh) is set to 1.0σ except for the [4Fe–4S] region in Cluster 52, where it is set to 1.5σ. The contour level for the *F*_o_ − *F*_c_ map is set to 3.0σ (green mesh, positive; red mesh, negative) in all figures. Only the main chain is depicted in the N-terminal region: ‘unfolded’ (purple) and ‘folded’ (blue). The variable N-terminal region (Ser7–Tyr12) was omitted and the occupancy for the [4Fe–4S] cluster was set to 1.0 in map calculation. The figures were generated by *Coot*.

**Figure 10:**
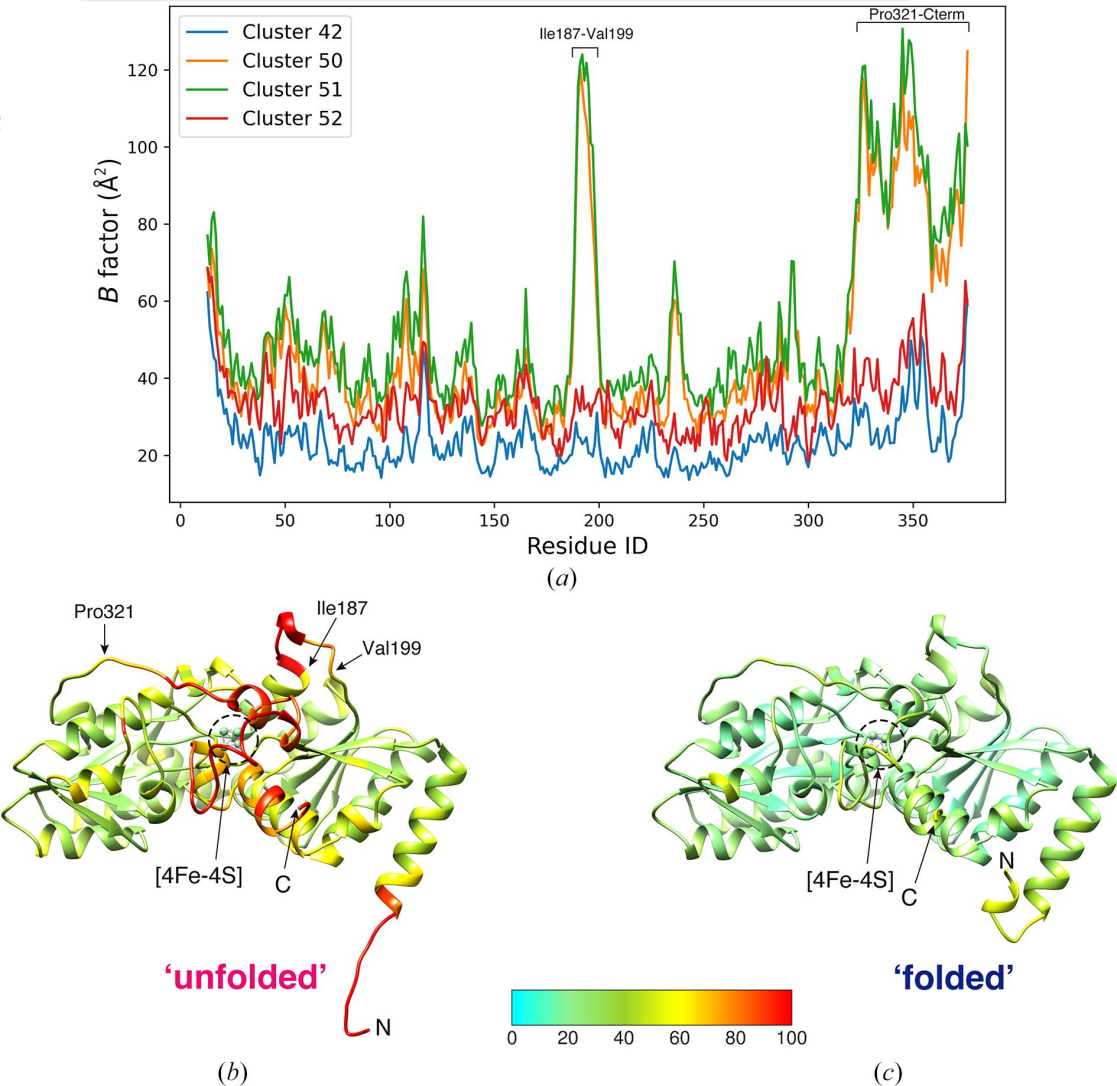
*B*-factor analysis of *Aa*HypD-C360S structures obtained from different clusters in intensity-based HCA. (*a*) Plot of the *B* factors of C^α^ atoms in data from different clusters. (*b*, *c*) Main-chain trace of *Aa*HypD-C360S with an ‘unfolded’ N-terminus obtained from Cluster 50 (*b*) and with a ‘folded’ N-terminus obtained from Cluster 52 (*c*). *B*-factor-based coloring was applied using *Coot*. The N-terminus and C-terminus are depicted in the figure as ‘N’ and ‘C’, respectively. The structure with the ‘unfolded’ N-terminus showed a significantly high *B* factor around the [4Fe–4S] cluster. The figures were generated by *UCSF Chimera* (Pettersen *et al.*, 2004 ▸).

The values of the isomorphic threshold were about 1.9–2.2 based on the dendrogram in Fig. 8 ▸, and Clusters 61, 63 and 64 were polymorph candidates in terms of this threshold. Since the Ward distance was very large compared with the other samples in this paper, we decided to carefully perform structural analysis on the clusters for which complete data sets were available and compare the details of this sample. Among the data from these clusters, some polymorphs that have differences around the N-terminal and [4Fe–4S] regions were identified by examining the corresponding electron-density maps (Fig. 9 ▸ and Supplementary Fig. S18). Significant differences were found in Clusters 50 and 52. In Cluster 50, the N-terminal region was unfolded (referred to as the ‘unfolded’ conformation) with no secondary structure and the occupancy of the [4Fe–4S] cluster decreased with disorder of the surrounding area (Figs. 9 ▸ *a* and 9 ▸ *b*). In contrast, in Cluster 52 the N-terminal region was folded towards the protein side (referred to as the ‘folded’ conformation) and the surrounding region around the [4Fe–4S] cluster was well ordered (Figs. 9 ▸ *c* and 9 ▸ *d*). The electron-density map in Cluster 51 (Supplementary Figs. S18*a* and S18*b* ) exhibited a similar trend in Cluster 50. However, the negative *F*_o_ − *F*_c_ peak of the [4Fe–4S] cluster was decreased in Cluster 51. The electron-density map in Cluster 42 was similar to that in Cluster 52 (Supplementary Figs. S18*c* and S18*d* ). However, the ‘folded’ N-terminal region was more evident in Cluster 52. Two different N-terminal conformations were strongly related to disorder around the [4Fe–4S] cluster. *B*-factor analysis clearly indicated that an ‘unfolded’ conformation in the N-terminus destabilized the [4Fe–4S] surrounding region (Fig. 10 ▸). From data in Cluster 54, a solution with a significantly high *R*_free_ (>0.4) was obtained. In Cluster 47, complete data were not obtained because most chunks were rejected by outlier rejection in *KAMO*. In this example, the most significant structural differences (the N-terminal and [4Fe–4S] regions) appeared to be separated at the first branch of the dendrogram. During further separation, more slight differences (occupancies) seemed to be classified (Clusters 50 and 51 or Cluster 52 and 42).

Consequently, Clusters 61, 63 and 64, classified from our ‘isomorphic threshold’, are the parent nodes of the data where the characteristic structures were found. Cluster 61 is the parent node for Clusters 50 and 51, Cluster 63 is the parent node for Cluster 52, and Cluster 64 is the parent node for Cluster 42 (Fig. 8 ▸). Considering only locally for Cluster 61, *W*_0_ is 1.0, corresponding to an isomorphic threshold of 0.6–0.7. This allows Clusters 50 and 51 to be considered as classifiable within this cluster. Our isomorphic thresholds also proved to be valid for this sample. Polymorphs were also found in crystals obtained under the same crystallization conditions. This result indicates that a protein may have several metastable conformations even under identical crystallization conditions. The results of this study indicate that when the absolute value of the Ward distance on the dendrogram is large, as in the present sample, it is useful to try to analyze the structure of subclusters of nodes that diverged at the isomorphic threshold.

The median values of CC distributions for homologous data pairs in Clusters 50, 51, 52 and 42 were 0.976, 0.961, 0.935 and 0.966, respectively. The corresponding standard deviations were 0.019, 0.019, 0.020 and 0.019, respectively. These statistics are filtered by *d*_CC_ < 0.4 as for the other samples. As expected from the resultant dendrogram (Fig. 8 ▸), there are significant CC changes between the data pairs from Cluster 50 or 51 and Cluster 52 or 42. Even for the CC distribution for the data pairs between Cluster 50 and Cluster 51, where the smallest difference was found from the dendrogram, the mean value of the CC distribution was 0.946 and the corresponding standard deviation was 0.020.

### 3.3. Current limitations of intensity-based HCA and conceivable best practices for further applications

The results for the two representative samples demonstrated that intensity-based HCA with the proposed ‘isomorphic threshold’ can be a useful indicator to detect polymorphs in data sets obtained from multiple crystals. In addition, splitting the data collected by a helical scheme into several chunks could be beneficial for the analysis of structural polymorphs within the same crystals. Even for well-known proteins whose structures have already been determined, structural polymorphs may possibly have been overlooked. The scope of this guideline is intended for cases in which multiple crystals of 50 µm or larger are used such that complete data can be collected from a single crystal with slight variation in unit-cell constants. We focus on finding polymorphs by collecting large wedge data sets of 360° or more through helical data collection from multiple large crystals and clustering the data into chunk data sets of 30° or more.

There is an explicit limitation for intensity-based HCA. A certain number of common reflections are required among the data sets to calculate the CC value. As a default setting in *KAMO*, at least three common reflections are required between each data set. In SWSX, diffraction data are collected with 5–20° rotation from each crystal, which results in fewer common reflections. The number of common reflections also decreases due to the lower resolution or crystallographic symmetry. In this study, the chunk size was set to 30°, where the number of common reflections is expected to be adequate.

To investigate the limitation on chunk size (rotation range), the numbers of common reflections and rejected data in different chunk sizes were plotted using the high-resolution trypsin data set from 0.5° to 30° (Supplementary Fig. S19). The number of chunks excluded from the CC calculation was drastically increased below 3°. When the chunk size was set to 0.5°, almost all data were rejected (99.0%). This result is consistent with a previous report on intensity-based HCA applied to serial synchrotron crystallography (SSX) data (2° per crystal) using *ccCluster* (Santoni *et al.*, 2017 ▸). Obviously, it is not realistic to apply intensity-based HCA to single-frame data with extremely narrow or no oscillation data, such as those collected by the serial femtosecond crystallography (SFX) or the serial synchrotron rotation crystallography (SSROX) approaches.

In addition, to investigate a sufficient number of common reflections for intensity-based HCA, the number of common reflections was evaluated in different chunk sizes for the data sets used in this study. Since the rejected data increased below 3° in the case of trypsin, log(number of common reflections) ≥ 2.5 seems promising. The fraction of common reflections in the total reflections is highly dependent on the crystallographic symmetry (Supplementary Fig. S20). If the resolution is relatively low or the crystallographic symmetry is not high, a large molecular weight could cover the number of reflections, as exhibited in the example of the Trn1–peptide complex. If possible, partial data collection using the helical scheme or a larger rotation range of data will help to increase the common reflections for polymorph analysis with intensity-based HCA.

Unit-cell-based HCA can still be useful when intensity-based HCA is not available. Even for single-frame data, such as SFX data, unit-cell constants are available. Therefore, unit-cell-based HCA can be applied to any given diffraction data set. In actual SFX data, data sets with different unit-cell properties are sometimes mixed into the entire data sets (Nomura *et al.*, 2021 ▸). Considering a general case, unit-cell-based HCA should first be applied to filter out the data set with different unit-cell parameters. If the variation in unit-cell constants is relatively small, say the largest LCV value is less than 1%, then it is likely that unit-cell-based HCA will not yield good classification results. However, polymorphs could be found from the results of unit-cell-based HCA. In a recent study (Soares *et al.*, 2022 ▸), the distance metric for unit-cell-based HCA has been improved. Furthermore, outlier rejection during the merging step as implemented in *KAMO* could be useful to reduce a contaminated data set.

Although we classified polymorphs by single-step intensity-based clustering, unit-cell parameters should be considered for more accurate classification in general cases. From the results of unit-cell-based HCA for trypsin data sets, chunks from the same data were separated into different branches (Figs. 1 ▸ and 3 ▸). If intensity-based HCA is further applied to the clusters obtained from unit-cell-based HCA, the number of data sets remaining in the final stage of data merging may decrease. Thus, single-step clustering is preferable when the total number of data sets is small. HCA with two-dimensional parameters, including both unit-cell parameters and intensity correlation, is under consideration for more effective single-step clustering.

## 4. Conclusions and outlook

Based on the investigations on the test cases, we propose that the ‘isomorphic threshold’ for classification by intensity-based HCA of several hundred 30° chunk data sets is the Ward distance in the top row of the dendrogram multiplied by 0.6–0.7. In the representative samples, polymorphs were successfully detected by intensity-based HCA with our suggested threshold. The scope of this guideline basically includes cases in which multiple crystals of larger than 50 µm are used and complete data can be collected from each single crystal with a slight variation in unit-cell constants. We focus on finding polymorphs by collecting large wedge data sets of 360° or more through helical data collection from multiple large crystals and clustering them into chunk data sets of 30° or more.

The results of this study, including standard and representative samples, show that single-step intensity-based HCA with the proposed ‘isomorphic threshold’ is effective for the detection of polymorphs using multiple data sets. Although helical data collection and HCA have been used in MX experiments and analyses, we demonstrated several advantages consistent with current HDRMX trends. The helical data scheme is unique in that it can be applied to polymorph analysis within crystals by dividing the complete data set into chunks, as the data are collected while translating the position of X-ray exposure. Indeed, our results suggest the possibility of the presence of polymorphs within crystals. The continuous helical data collection adopted in the *ZOO* automated data-acquisition system at SPring-8 collects data from each crystal volume with a uniform dose, which may reduce structural inhomogeneities due to radiation damage compared with single-point rotation. Even when multiple crystals are used, the isomorphism among crystals due to radiation damage can be suppressed to the same extent, allowing discussion of only the structural differences that exist in the crystals. In addition, highly efficient automated data collection increases the number of data sets that can be measured in a given time, allowing the analysis of many polymorphs with higher resolution.

Our findings could be widely applied to other protein samples. Even though the resolution was relatively low (approximately 4 Å), physiologically meaningful polymorphs can be identified, as exhibited in the example of the Trn1–peptide complex. The representative examples also showed that protein molecules may exhibit different structures within the same crystal. In addition, polymorphs can be found from multiple crystals obtained using the same crystallization conditions in both practical cases. Determination of the ‘isomorphic’ threshold enables polymorph detection, even if the existence of polymorphs was not expected or detected during sample preparation. It will also facilitate broader applications by automating the polymorph analysis with our proposed ‘isomorphic’ threshold.

The suggested polymorph analysis could help to obtain various structural snapshots during the functional process of the protein to elucidate the molecular mechanism. The determination of multiple structural snapshots will also contribute to more accurate structure prediction, as realized by *AlphaFold*2 (Jumper *et al.*, 2021 ▸) and *RoseTTAFold* (Baek *et al.*, 2021 ▸). However, structural information on time series or reaction pathways among the identified polymorphs is not available using our approach. It may be complemented by molecular-dynamics (MD) simulations. For instance, analysis of the free-energy landscape will help in understanding the dynamic structural mechanism during protein function (Oide *et al.*, 2020 ▸). Polymorph analysis will be further enhanced along with techniques for inducing structural change, such as ligand mixing. Although time-resolved crystallography is a powerful tool for the analysis of dynamics, the proposed polymorph analysis will help to compensate for numerous difficulties in controlling such reactions. Expanding intensity-based HCA to single-frame data (from SFX or SSROX) should also be developed in the near future.

## 5. Availability

The analysis presented here can be performed anywhere by installing the program *KAMO*, which is available from GitHub (https://github.com/keitaroyam/yamtbx). The raw diffraction data used in this study are available from Zenodo. The links are https://doi.org/10.5281/zenodo.7067666 for apo-trypsin; https://doi.org/10.5281/zenodo.7068055 for benzamidine-bound trypsin; https://doi.org/10.5281/zenodo.7067758 for tryptamine-bound trypsin; https://doi.org/10.5281/zenodo.7068185 for HypD and https://doi.org/10.5281/zenodo.7067871 for Trn1.

## Supplementary Material

Supporting information including Supplementary Figures and Tables. DOI: 10.1107/S2059798323007039/wa5143sup1.pdf

Raw diffraction images of apo-trypsin: https://doi.org/10.5281/zenodo.7067666

Raw diffraction images of 4-methoxybenzamidine-bound trypsin: https://doi.org/10.5281/zenodo.7068055

Raw diffraction images of 5-chlorotryptamine-bound trypsin: https://doi.org/10.5281/zenodo.7067758

Raw diffraction images of [NiFe]-hydrogenase maturation factor HypD from Aquifex aeolicus (C360S mutant): https://doi.org/10.5281/zenodo.7068185

Raw diffraction images of transportin-1 in complex with a nuclear localization signal peptide: https://doi.org/10.5281/zenodo.7067871
